# The Expression and Regulation of Chemerin in the Epidermis

**DOI:** 10.1371/journal.pone.0117830

**Published:** 2015-02-06

**Authors:** Magdalena Banas, Aneta Zegar, Mateusz Kwitniewski, Katarzyna Zabieglo, Joanna Marczynska, Monika Kapinska-Mrowiecka, Melissa LaJevic, Brian A. Zabel, Joanna Cichy

**Affiliations:** 1 Department of Immunology, Faculty of Biochemistry, Biophysics and Biotechnology, Jagiellonian University, Kraków, Poland; 2 Department of Dermatology, Zeromski Hospital, Kraków, Poland; 3 Stanford University School of Medicine, Department of Pathology, Stanford, California, United States of America; 4 Palo Alto Veterans Institute for Research, VA Palo Alto Health Care System, Palo Alto, California, United States of America; French National Centre for Scientific Research, FRANCE

## Abstract

Chemerin is a protein ligand for the G protein-coupled receptor CMKLR1 and also binds to two atypical heptahelical receptors, CCRL2 and GPR1. Chemerin is a leukocyte attractant, adipokine, and antimicrobial protein. Although chemerin was initially identified as a highly expressed gene in healthy skin keratinocytes that was downregulated during psoriasis, the regulation of chemerin and its receptors in the skin by specific cytokines and microbial factors remains unexplored. Here we show that chemerin, CMKLR1, CCRL2 and GPR1 are expressed in human and mouse epidermis, suggesting that this tissue may be both a source and target for chemerin mediated effects. In human skin cultures, chemerin is significantly downregulated by IL-17 and IL-22, key cytokines implicated in psoriasis, whereas it is upregulated by acute phase cytokines oncostatin M and IL-1β. Moreover, we show that human keratinocytes *in vitro *and mouse skin *in vivo *respond to specific microbial signals to regulate expression levels of chemerin and its receptors. Furthermore, in a cutaneous infection model, chemerin is required for maximal bactericidal effects *in vivo*. Together, our findings reveal previously uncharacterized regulators of chemerin expression in skin and identify a physiologic role for chemerin in skin barrier defense against microbial pathogens.

## Introduction

Chemerin, also known as tazarotene induced gene 2 (Tig2) or retinoic acid receptor responder protein 2 (RARRES2), is a broadly expressed leukocyte attractant ligand for serpentine, G protein-associated receptor CMKLR1 (chemokine-like receptor 1) [1,2,3]. CMKLR1+ plasmacytoid dendritic cells (pDCs), macrophages and NK cells are critical in bridging the innate and adaptive immune responses [3,4,5,6]. Chemerin is secreted as an inactive precursor protein (Chem163S, with number and capital letter referring to the terminal amino acid position and single amino acid code, respectively). Chem163S can be converted to chemotactically active isoforms, such as Chem157S, through posttranslational carboxyl-terminal processing by a variety of proteinases [2,7,8,9].

Interest in chemerin has surged in the last few years as chemerin, in addition to its chemotactic function, was reported to regulate adipocyte differentiation [10], angiogenesis [11], osteoblastogenesis [12], myogenesis [13], and glucose homeostasis [14,15,16].

In addition to CMKLR1, two additional heptahelical receptors, GPR1 and CC-motif chemokine receptor-like 2 (CCRL2), bind chemerin with low nanomolar affinities similar to CMKLR1 [17,18]. However, among these receptors only chemerin binding to CMKLR1 triggers cell migration, intracellular calcium mobilization, and β-arrestin2 association and receptor internalization, all features common to classical G protein-coupled receptors. In contrast, chemerin binding to GPR1 triggers β-arrestin2 association and receptor internalization: whether it triggers intracellular calcium signaling is unclear [2,17]. The *in vivo* function of GPR1 remains relatively obscure, although recent studies using GPR1-deficient mice implicate the receptor in regulating glucose homeostasis during obesity [19]. CCRL2 regulates chemerin concentrations by sequestering secreted chemerin, concentrating it on the cell surface and presenting it to adjacent CMKLR1+ cells [18,20,21].

Although fat tissue and liver have been confirmed by multiple groups as key sites of chemerin production [22] and possibly responsible for the high nanomolar chemerin levels found circulating in plasma [23], chemerin is also expressed at epithelial barriers, including skin epidermis [24,25,26,27]. There is regional variation in the distribution of chemerin in healthy and diseased skin. Whereas chemerin is produced by keratinocytes in healthy skin, it is markedly downregulated in the epidermis of patients suffering from the autoinflammatory skin disease psoriasis. In contrast, normal dermis contains little chemerin, but affected psoriatic dermis is a significant source of chemerin as determined by immunohistochemistry [24,27]. These findings suggest an association between skin dysfunction and altered chemerin levels. We and others have previously reported that chemerin likely contributes to pDC recruitment to lesional psoriatic skin [5,24,28]. In addition, in normal skin, specifically the epidermis, chemerin functions as a potent antimicrobial protein, where it embodies a quantitatively significant fraction of the anti-bacterial activity of cultured keratinocytes [25]. Despite its roles in host defense and the pathogenesis of skin disease, the mechanisms underlying chemerin expression in skin remain poorly defined. The only known regulator of chemerin expression in epidermis is the anti-psoriatic synthetic retinoid-tazarotene, which upregulates chemerin level in skin raft cultures [27].

Here we show that epidermal chemerin represents an important source of this protein in the skin under steady-state conditions and is significantly downregulated by cytokines implicated in psoriasis, whereas it is markedly upregulated by bacteria and acute phase mediators.

## Materials and Methods

### Materials

Human recombinant OSM, IL-1β, IL17 and IL22 were purchased from R&D Systems, whereas *E. coli-*derived LPS from Sigma-Aldrich. *S. aureus* ATCC 35556 and *E. coli* HB101 were obtained from DSMZ. Bacteria were grown in tryptic soy broth (TSB) (Sigma) to mid-logarithmic phase and used for subsequent experiments at 1x10^7^ colony-forming units (CFU). When indicated bacteria were heat-killed by incubation of 10^7^CFU bacteria/100 microl PBS at 85°C for 20 min., or were incubated with bacteriocidic concentration of ampicilin (1 μg/ml) for 24h/48h.

### Mice

Female or male 8–12 weeks old C57BL6 mice and chemerin-deficient mice on C57BL6 background, as well as WT Balb/C mice, CMKLR1KO [29], CCRL2KO [18] or double CMKLR1/CCRL2KO mice on Balb/C background were used in these studies. The chemerin KO mice used for this research project were generated by the trans-NIH Knock-Out Mouse Project (KOMP) and obtained from the KOMP Repository (www.komp.org). NIH grants to Velocigene at Regeneron Inc (U01HG004085) and the CSD Consortium (U01HG004080) funded the generation of gene-targeted ES cells for 8500 genes in the KOMP Program and archived and distributed by the KOMP Repository at UC Davis and CHORI (U42RR024244). Mice were housed under pathogen-free conditions in the animal facility at the Faculty of Biochemistry, Biophysics and Biotechnology of Jagiellonian University or the Veterans Affairs Palo Alto Health Care System. Liver, white adipose tissue (WAT) and skin were harvested and subjected to RT-QPCR or ELISA analysis. Blood was collected in EDTA coated tubes and centrifuged at 2000g for 6 min. Collected plasma was then subjected to ELISA analysis. This study was carried out in strict accordance with the recommendations in the Guide for the Care and Use of Laboratory Animals of the National Institutes of Health. The protocols 119/2010, 149/2013 and A3088-01 were approved by the First Local Ethical Committee on Animal Testing at the Jagiellonian University in Krakow or the Institutional Animal Use and Care Committee at the Veterans Affairs Palo Alto Health Care System (AAALAC-accredited facility). All surgery was performed under ketamine/xylazine anesthesia, and all efforts were made to minimize suffering. Mice were sacrificed by inhalation of CO2.

### Topical skin infection

Mice were anesthetized and a small dorsal area of the skin was shaved and sterilized with ethanol. The shaved area was punctured six times at two places using a syringe needle (BD Microlance, 0.3 × 19 mm) (MidMeds). Two rubber rings both with 8-mm inner diameter were subsequently attached using an ethylcyanoacrylate-based adhesive and the rings were covered with OpSite (Medisave). 1 × 10^7^ CFU of *S. aureus* or *E. coli* in a volume of 50μl (PBS) was thereafter injected through the OpSite into the cavity formed by the rubber rings. The ring injected with sterile PBS was used as control. Mice were killed after 24h and the skin within the side of the rings was retrieved for RT-QPCR and ELISA analysis as well as enumeration of CFU.

### Cell culture

All human studies were performed in compliance with ethical protocols KBET/44/B/2011 and KBET/87/B/2014 approved by the Jagiellonian University Institutional Bioethics Committee. Declaration of Helsinki protocols were followed. All participants provided their written informed consent to participate in these studies as recommended by the ethical board. Normal human keratinocytes were isolated from excess skin from donors obtained at the time of cosmetic surgery for mole removal or during plastic surgery. Donors included 23 healthy individuals (age 36±18 years; F:M, 10:13). Skin biopsies were rinsed three times in calcium- and magnesium-free PBS supplemented with penicillin (5000U/ml) – streptomycin (5mg/ml) (all from Sigma). After washing, the biopsy was placed in PBS containing dispase (12U/mL, Gibco) for 16h at 4°C. Next, the epidermis was separated from the dermis with forceps followed by treatment with 0.05% trypsin with 2 mM EDTA (Sigma) to isolate epidermal cells. Cells were cultured in serum free KGM-Gold medium (Lonza Group Ltd.) to generate passage 1 cells. The keratinocytes were then plated at density of 1×10^5^ cells per well on permeable inserts (12-mm-diameter, 0.4μm pore size; Millipore, Millicell culture inserts) in PCT Epidermal Keratinocyte Medium (CellnTec). Cells were cultured at 37°C in presence of 5% CO_2_ until confluence. Polarized skin structures that resemble in vivo stratified epidermis were generated by air-liquid interface cultures grown in 3D Prime Medium (CellnTec) for 11 days. Cells were than treated with the indicated factors for 24h or 48h. The final concentration of stimulating factors were based on previous publications [30,31,32], or for LPS optimized experimentally [from 10ng to 10 μg/ml], and were as follows; OSM 50ng/ml, IL1 10ng/ml, IL17 200ng/ml, IL22 200ng/ml and LPS 1μg/ml.

### Preparation of skin homogenates and epidermis lysates

Skin was homogenized at 100 mg/ml in water containing protease inhibitor (Complete, Roche) or lysed as described for the epidermis. The epidermis was separated from the dermis as described above. Epidermis was then lysed in a RIPA buffer (25mM Tris-HCl, pH 7.6, 150mM NaCl, 1% NP-40, 1% sodium deoxycholate, 0.1% SDS) containing protease inhibitors, passed through a 40 μm cell strainer and incubated o/n at 4°C. Extracts were centrifuged at 10,000g for 30 min to remove cellular debris and then normalized based on protein concentration as determined by BCA assay (Sigma). Lysates were stored at -20°C until used.

### RT-QPCR

Total RNA was extracted as previously described [33] and converted to cDNA using NxGen M-MulV reverse transcriptase (Lucigen) with random primers (Promega). Real time PCR was performed on the 7500Fast (Applied Biosystems) using SYBR Green I containing universal PCR master mix (A&A Biotechnology) and primers specific for; human chemerin (5’ TGGAAGAAACCCGAGTGCAAA-3’, 5’-AGAACTTGGGTCTCTATGGGG), CMKLR1 (5’- ATGGACTACCACTGGGTTTTCGGG-3’, 5’-GAAGACGAGAGATGGGGAAACTCAAG-3’), CCLR2 (5’-CCGTTTCTTAAAAGGCAGTCTGAA-3’, 5’-GTCATACTTGTCACATTGCTCTGC-3’), GPR1 (5’-AATGCCATCGTCATTTGGTT-3’, 5’-CAACTGGGCAGTGAAGGAAT-3’), GAPDH (5’- GAGTCAACGGTTTGGTCGTATTG-3’, 5’- ATGTAGTTGAGGTCAATGAAGGGG -3’) and beta-2-microglobulin (B2M) (5’- TCAGCAAGGACTGGTCTTTCTATC-3’, 5’- GCTTACATGTCTCGATCCCACTTA-3’), as well as mouse chemerin (5’- CTTCTCCCGTTTGGTTTGATT-3’, 5’- TACAGGTGGCTCCTCTGGAGGAGT-3’), CMKLR1 (5’-CAAGCAAACAGCCACTACCA-3’, 5’-TAGATGCCGGAGTCGTTGTAA- 3’), CCLR2 (5’- TTCCAACATCCTCCTCCTTG -3’, 5’- GATGCACGCAACAATACCAC -3’), GPR1 (5’- AAAAGCTGTTTGAGGCTAGAAAGG -3’, 5’- AGGAAATCTGTTAATGTTCTGTGCG -3’), cyclophilin (5’- AGCATACAGGTCCTGGCATCTTGT -3’, 5’- CAAAGACCACATGCTTGCCATCCA -3’) and ribosomal protein L13a (RPL13A) (5’-CCTCAAGGTGTTGGATGGGAT-3’, 5’- GTAAGCAAACTTTCTGGTAGGCTTC-3’). The Excel based application Best-Keeper was used to analyze the expression stabilities of the commonly used reference genes [34]. Based on this analysis, murine cyclophylin A and RPL13A, or human B2M and GAPDH were selected as housekeeping genes for normalizing RNA expression in RT-QPCR. Relative gene expression normalized to geometric mean of these housekeeping genes was calculated using the 2^-ΔΔCT^ method [35,36]. Whenever possible, specificity of PCR products was verified using KO mice.

### ELISA

Chemerin levels in conditioned media or in epidermis lysates was quantified by human or mouse-specific ELISA. High-binding ELISA strips (Nunc) were coated with mouse-anti-human chemerin mAbs (MAB23241), or goat-anti-mouse Abs (AF2325) (both from R&D Systems) in Tris-buffered saline (50 mM Tris-HCl pH 9.5, 150 mM NaCl). The plates were then washed with PBS containing 0.1% Tween 20, and nonspecific protein-binding sites were blocked with 3% BSA in PBS. Human or mouse recombinant chemerin was used as a standard. Chemerin was detected using biotin-conjugated goat anti-human chemerin Abs (BAF2324) or biotin-conjugated rat-anti mouse chemerin mAbs (BAM2325) followed by streptavidin-HRP (BD Science). The reaction was developed with TMB substrate (BD Science).The ELISA detects both the 163S and 157S chemerin. Alternatively, chemerin in mouse skin homogenates (100%) and plasma samples (diluted 1/200) was detected by comercially available ELISA (R&D Systems), according to the manufacturer’s instructions. The levels of chemerin in plasma or skin homogenates and epidermis extracts were undetectable in chemerin KO mice.

### Immunohistochemistry

Epidermal tissues were fixed in 4% formaldehyde and embedded in paraffin. Paraffin 6-μm sections were then prepared from keratinocyte cultures. Sections were blocked with goat IgG and stained with the rabbit anti-human chemerin (H-002-52 Phoenix Pharmaceuticals) or control IgG (normal rabbit IgG, Jackson Immunoresearch) followed by APC-goat anti-rabbit IgG F(ab)2 (Jackson Immunoresearch). Blocking and staining were performed in the presence of 0.1% saponin. The sections were counterstained with Hoechst 33258 (Invitrogen). Images were captured with a fluorescence microscope (NIKON, Eclipse) and analyzed by NIS elements software (Nikon).

### Statistical analysis

For statistical evaluation, one way ANOVA followed by a Bonferroni post hoc test, or two-tailed Student’s *t* test was performed.

## Results

### Expression of chemerin and its receptors in normal skin

**Fig 1:**
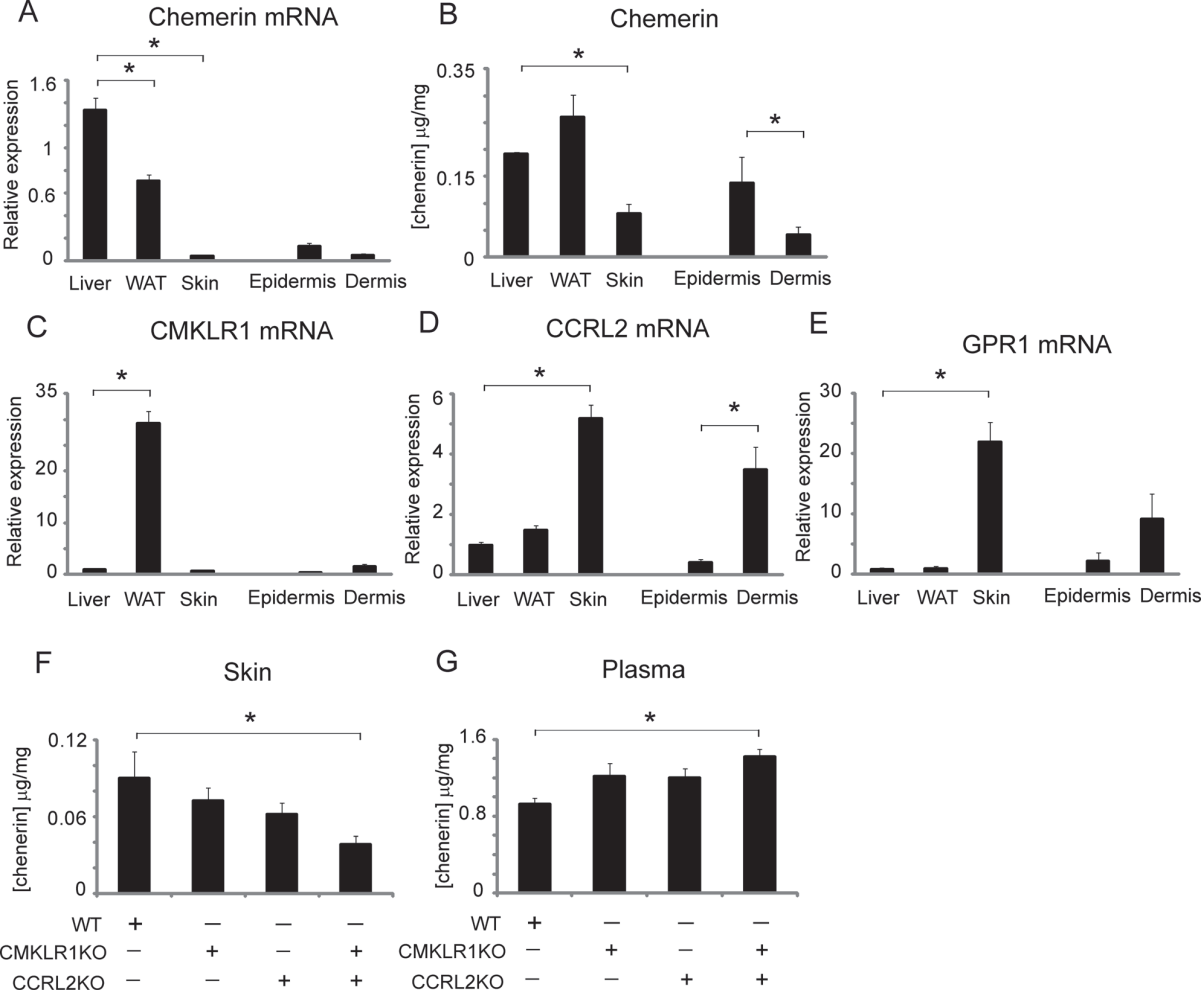
Chemerin and chemerin receptor expression in mouse skin. Chemerin mRNA expression (A), chemerin protein expression in tissue lysates (B), chemerin receptor (CMKLR1, CCRL2, GPR1) mRNA expression (C-E), or chemerin protein expression in skin homogenates (F) and chemerin expression in plasma (G) was measured in the indicated tissues isolated from C57BL/6 mice (A-E) or the indicated mice on BalbC backround (F-G) by RT-QPCR and ELISA. The expression data of the indicated genes was normalized to cyclophilin A and RPL13A, and presented relative to liver as the mean ± SEM, n = 4–5 different mice (A, C-E). The amount of chemerin protein in plasma, or skin lysates and homogenates normalized to total protein is shown as the mean ± SEM, n = 4 (B), or n≥6 mice (F-G). Statistical significance is indicated by asterisk(s); *p<0.05, **p<0.01, by ANOVA followed by a Bonferroni post hoc test.

Under normal conditions, expression of chemerin mRNA in skin was approximately ten-fold and six-fold lower compared to liver and white adipose tissue (WAT), respectively (Fig. 1A). On the other hand, chemerin protein levels in tissue lysates were only two-fold and three-fold lower compared to liver and WAT, respectively (liver: 190±40 ng/mg total protein; WAT: 267±37 ng/mg; skin: 86±17 ng/mg) (Fig. 1B). When the skin was split into epidermal and dermal sheets, chemerin was found primarily in the epidermis (Fig. 1A and B), in agreement with previous immunohistochemistry results [26], suggesting that chemerin mRNA and protein levels in total skin might be diluted by low expression of chemerin in dermis. Notably, chemerin protein levels in epidermal isolations (133±41 ng/mg of total protein) were similar to the levels detected in the liver.

Since chemerin protein levels in tissue lysates might be affected by binding of secreted chemerin to chemerin receptors [22], we next analyzed expression of CMKLR1, CCRL2, and GPR1. Although mRNA for all three receptors was present in liver, WAT and skin, CMKLR1 was expressed most highly in WAT, whereas CCRL2 and GPR1 were expressed most highly in skin (Fig. 1C-E). CMKLR1and GPR1 expression tended to be higher in the dermal compartment compared with epidermal layers, and was significantly higher for CCRL2. If CMKLR1 and CCRL2 serve as chemerin receptors in skin, then skin chemerin levels may be diminished in the absence of these receptors. As demonstrated in Fig. 1F, skin chemerin levels tended to be lower in CMKLR1 KO and CCRL2 KO mice and were the lowest in mice with a combined deletion of CMKLR1 and CCRL2 (CMKLR1/CCRL2 KO) compared to WT mice. On the other hand, plasma chemerin levels showed the opposite trend and were highest in CMKLR1/CCRL2 KO mice (Fig. 1G). This is consistent with a previous report indicating elevated chemerin levels in CCRL2 KO mice [21] and is a general phenomenon common to cognate receptor-deficient mice [37]. Together, these data suggest that chemerin is sequestered in skin by CMKLR1 and CCRL2.

**Fig 2:**
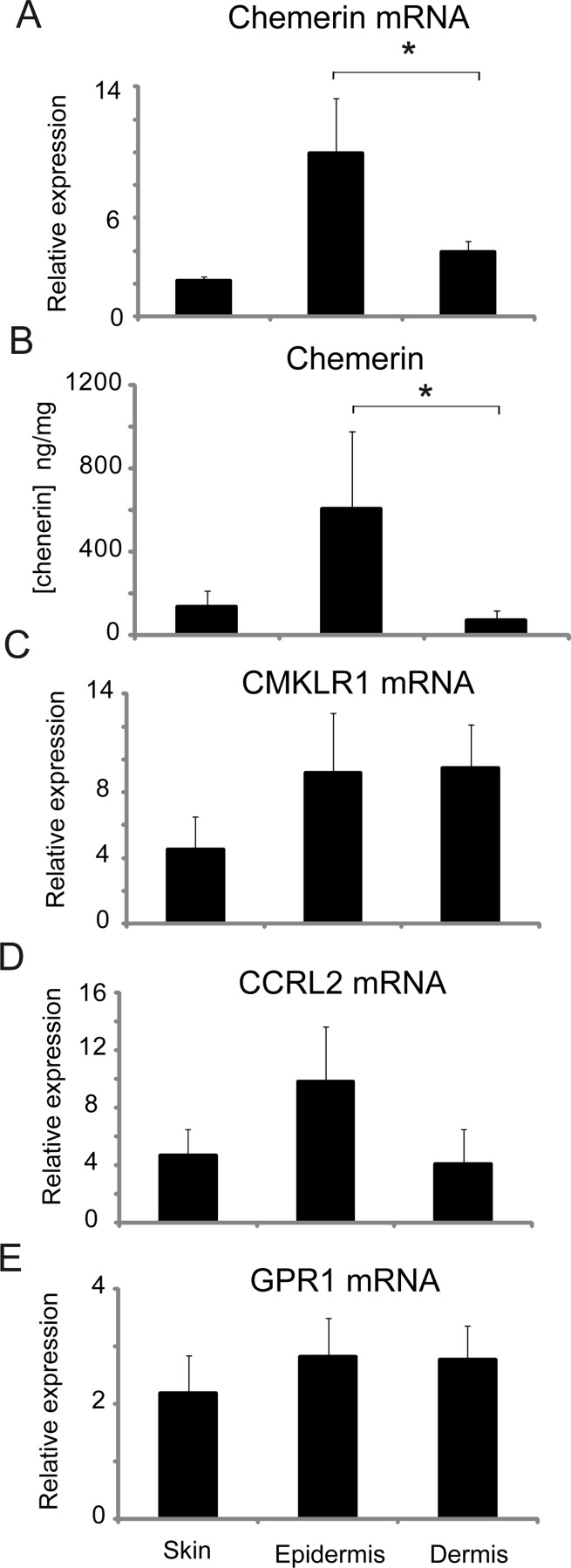
Chemerin in healthy human skin is primarily expressed in the epidermis. Chemerin mRNA expression (A), chemerin protein expression in tissue lysates (B), and chemerin receptor (CMKLR1, CCRL2, GPR1) mRNA expression (C-E), was measured in the indicated tissues isolated from healthy human donors. Total RNA was subjected to RT-QPCR. The expression data of the indicated genes was normalized to B2M and GAPDH, and presented relative to skin (A, C-E). The amount of chemerin in skin lysates, normalized to total protein was determined by ELISA (B). The mean of n = 6 (A, C-E) or n = 8 (B) different donors ± SEM is shown. Statistical significance between epidermis and dermis is shown by asterisk(s); *p<0.05, **p<0.01, by ANOVA followed by a Bonferroni post hoc test.

We next evaluated chemerin and chemerin receptor levels in human skin. Similar to mouse skin, chemerin levels were significantly higher in human epidermis compared with dermis (Fig. 2A and B). On the other hand, there were no significant differences in human epidermal vs. dermal expression of CMKLR1, CCRL2 and GPR1 (Fig. 2C-E).

### Regulation of chemerin expression in keratinocytes by acute phase mediators

**Fig 3:**
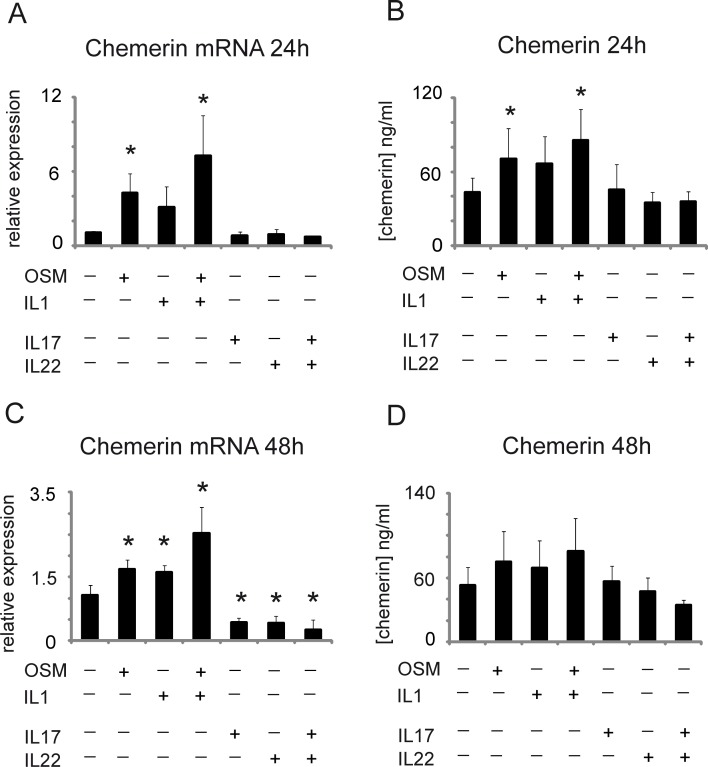
Psoriasis-associated cytokines downregulate chemerin and acute phase cytokines upregulate chemerin expression in epidermis. Normal keratinocytes grown in 3D culture were treated with the indicated factors for 24 (A-B) or 48h (C-D). Total RNA was subjected to RT-QPCR. Relative expression of stimulated cells over control is shown as the mean ±SD from five-nine independent experiments (A, C). Levels of secreted chemerin were determined in parallel in conditioned media by ELISA. Data show the mean ±SD from five-nine independent experiments (B, D). Statistical significance between control and the treated cells is shown by asterisk; *p<0.05 by ANOVA followed by a Bonferroni post hoc test.

**Fig 4:**
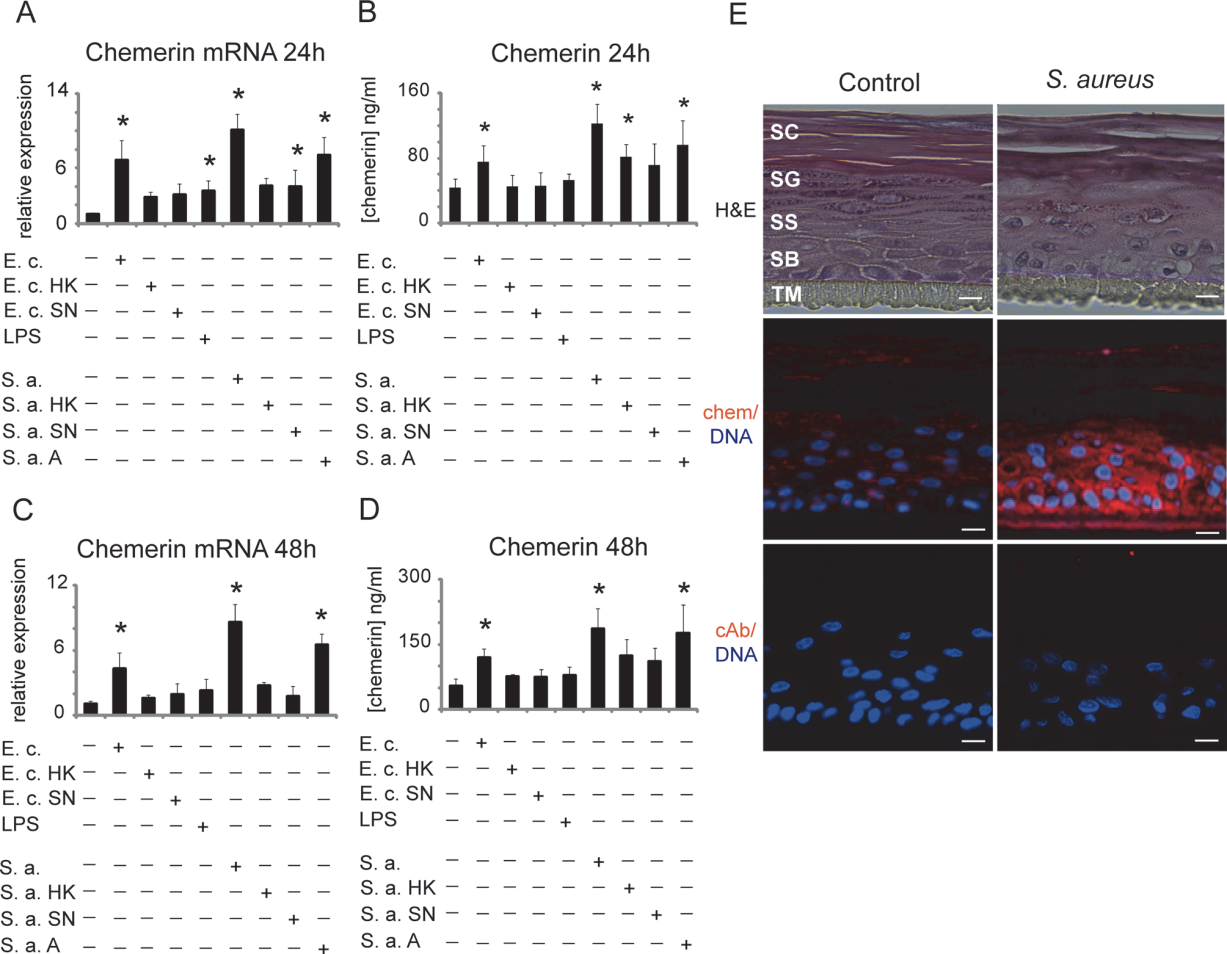
Bacteria upregulate chemerin expression in epidermis. Keratinocytes were treated with the indicated factors for 24 (A, B, E) or 48h (C, D). Total RNA was subjected to RT-QPCR. Relative expression of stimulated cells over control is shown as the mean ±SD from five-nine independent experiments (A, C). Levels of secreted chemerin were determined in parallel in conditioned media by ELISA. Data show the mean ±SD from five-nine independent experiments (B, D). Statistical significance between control and the treated cells is shown by asterisk; *p<0.05 by ANOVA followed by a Bonferroni post hoc test. E. c., *E. coli*; HK, heat-killed; SN, supernatant; S. a., *S. aureus*; A, ampicillin-treated. Microscope images of keratinocytes stained with hematoxilin and eosin (H&E) and fluorescence microscope images of keratinocytes stained for chemerin (chem) or control rabbit Abs (cAb) (red), with Hoechst counterstain to detect cell nuclei (blue). Scale bar = 10 μm. Data are representative of three different donors. SC, stratum corneum, SG, stratum granulosum, SS, stratum spinosum, SB stratum basale, TM transwell membrane (E).

To facilitate our studies investigating the mechanisms regulating chemerin expression in skin we generated pseudo-stratified, highly differentiated human epidermal tissue *in vitro*. In contrast to keratinocyte monolayers that do not fully recapitulate the multilayered differentiation of epidermis and express little-to-no chemerin ([27] and data not shown), this 3D tissue closely resembles the epidermis, and keratinocytes in these 3D cultures express high levels of chemerin Figs. 3 & 4. Importantly, the polarized nature of skin keratinocytes in this model allows for the anatomical segregation of epidermal responses. We applied cytokines to the basolateral compartment to mimic epidermal cytokine exposure resulting from immune cells infiltrating the skin [38,39,40,41].

We first asked if local chemerin synthesis in the skin was induced by acute phase mediators such as oncostatin M (OSM) and IL-1β, which mobilize protective acute phase reactants. Cells and conditioned media from cultured human skin equivalents were collected 24–48h after basolateral treatment, since the effect of OSM on gene expression is typically most profound at these time points [31,42,43,44]. Treatment with OSM, IL-1β, and the combination resulted in either a tendency to higher chemerin levels or statistically significant upregulation of chemerin mRNA and protein at both time points (Fig. 3). Chemerin production was the highest in response to OSM + IL-1β at 24h (7.3-fold increase over control by RNA analysis, and 2-fold by secreted protein analysis), suggesting additive effects (Fig. 3).

### Regulation of chemerin expression in keratinocytes by “psoriatic cytokines”

IL-17 and IL-22 drive keratinocyte pathology in psoriasis [39,40,41]. We next asked if IL-17 and Il-22 applied to the basolateral compartment affected chemerin expression/secretion in the epidermis model. IL-17 and IL-22 were equally efficacious in downregulating chemerin expression at 48h (on average 2.5-fold compared with untreated controls), and when used together exhibited an additive effect (4.3-fold reduction). Consistent with IL-17- and IL-22-mediated inhibition of chemerin RNA expression, secreted protein tend to be diminished (Fig. 3C and D). Together, these data suggest that chemerin is a regulatory target of IL-17 and IL-22 in epidermal tissue.

### Regulation of chemerin expression in human keratinocytes and mouse skin by bacteria

Since chemerin has antimicrobial activity in normal human skin, we next asked if its expression was modulated by bacteria exposure in the epidermal model (apical side treatment). We selected two bacteria strains, *E. coli* and *S. aureus*, both of which are susceptible to chemerin-dependent killing, although with different potencies (MIC = 3.1–6.3μg/ml vs. 12.5μg/ml for *E. coli* and *S. aureus*, respectively) [25]. *E. coli* markedly upregulated chemerin RNA expression (~7-fold), (Fig. 4A) and secreted protein (75±20 ng/ml versus 43±12 ng/ml in untreated cultures) at 24h (Fig. 4B). The effect of *E. coli* remained significant although somewhat diminished by 48h (Fig. 4C and D). Interestingly, compared with live bacteria, heat-killed counterparts triggered no significant effects on chemerin expression or secretion. This may be attributed to the ability of live microorganisms to replicate and/or express specialized stimulating factors. At least part of the stimulatory effect of *E. coli* was mediated by soluble factors, most likely LPS, as LPS alone significantly increased chemerin mRNA at 24h. Compared with *E. coli, S. aureus* was more effective in boosting chemerin expression, resulting in 10-fold and 8-fold induction of chemerin mRNA levels at 24h and 48h, respectively (Fig. 4A and C). The effect of *S. aureus* on chemerin gene expression was reflected in secreted chemerin protein levels, which increased from 43±12 to 123±24 ng/ml following 24h co-incubation, and from 53±17 to 185±47 ng/ml after 48h incubation with the bacteria (Fig. 4B and D). We hypothesized that since chemerin is more potent in killing *E. coli* compared with *S. aureus*, the keratinocytes in the in vitro culture may have been exposed to lower doses of *E. coli* than *S. aureus*. This may in turn result in the appearance of a more robust induction of chemerin by *S. aureus*. To address this, we treated *S. aureus* with bacteriostatic doses of ampicilin. This treatment resulted in comparable numbers of CFU for *E. coli* and *S. aureus* during their incubation with keratinocytes, and did not significantly change the chemerin levels in control keratinocytes (data not shown). There was no significant difference in epidermal chemerin induction by *S. aureus* vs. *S. aureus* treated with ampicillin, both of which were more effective than *E. coli* (Fig. 4A-D). These data imply that a *S. aureus*-intrinsic component stimulates higher levels of chemerin expression than *E. coli*, and that the effect is unrelated to differential microbe-specific killing potencies of chemerin. In addition, live *S. aureus* was ~3-fold more effective than heat-killed counterparts in inducing chemerin (Fig. 4A and C).

Since the effect of live *S. aureus* on chemerin expression dominated over other factors, we investigated this effect more closely by immunohistochemistry. *S. aureus*-treated and untreated skin equivalents appeared similar by microscopic analysis of H&E stained sections (Fig. 4E). Chemerin was present in all strata, with the exception of stratum granulosum. However, chemerin staining was more intense in all epidermal strata in *S. aureus*-treated skin vs. control, most notably in the stratum basale, suggesting that the elevated chemerin protein levels detected in conditioned media likely result from its secretion primarily by proliferating keratinocytes (Fig. 4E).

### Regulation of CMKLR1, CCRL2 and GPR1 expression in human epidermis equivalents

**Fig 5:**
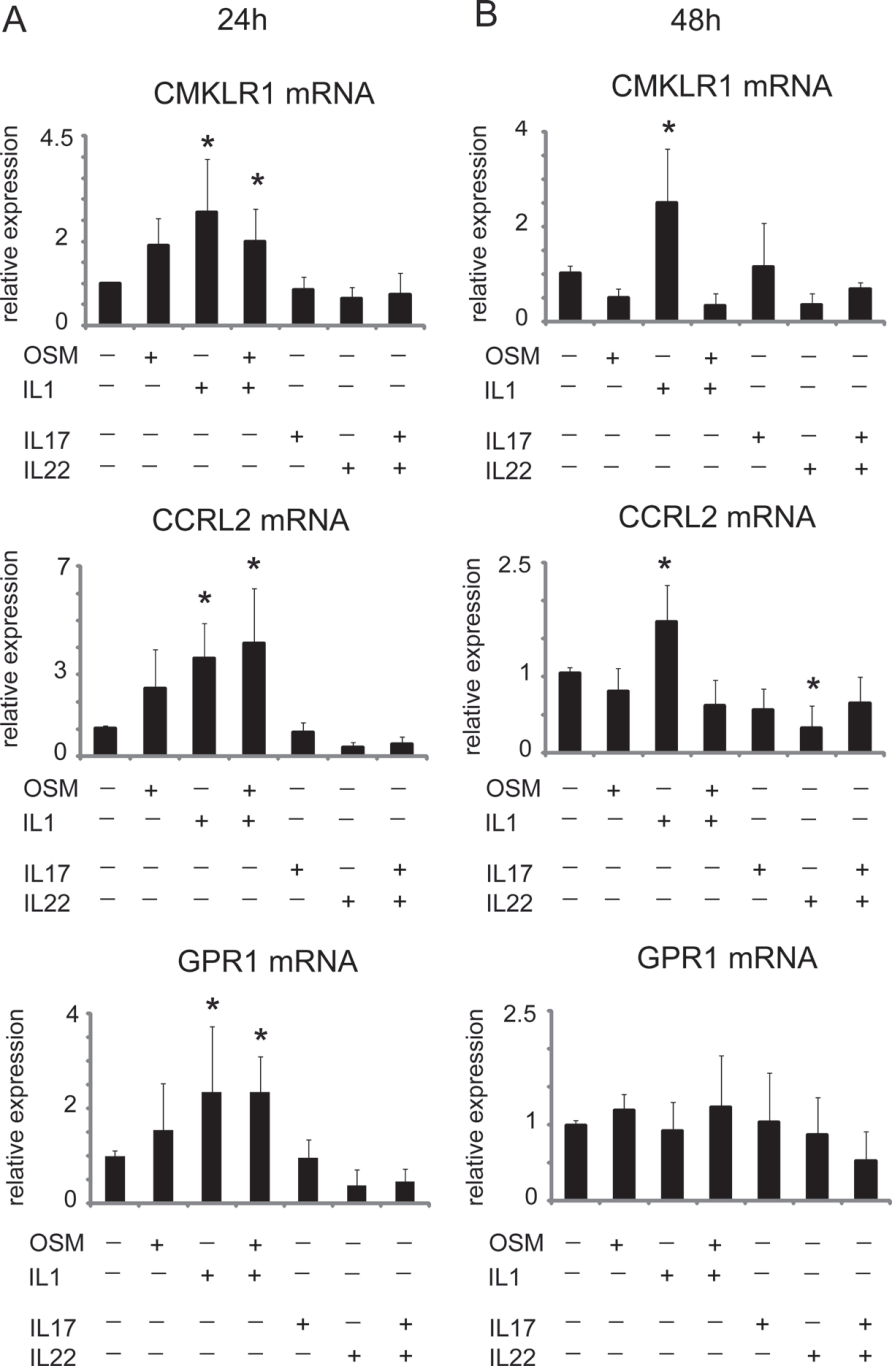
Expression of chemerin receptors in human skin equivalents treated with cytokines. Keratinocytes were treated with the indicated factors for 24h (A) or 48 h (B). RT-QPCR was performed and the expression data were normalized to cyclophilin A and expressed relative to unstimulated cells. Mean ± SD of 5–8 independent experiments is shown. Statistical significance comparing cytokine-treated cells vs. untreated cells (* p<0.05) was determined by ANOVA followed by a Bonferroni post hoc test.

We next evaluated the expression of chemerin receptors in skin equivalents in response to cytokines and bacteria. CMKLR1 mRNA levels were significantly upregulated following 24h treatment with IL-1β and the combination of IL-1β and OSM, while IL-17 and/or IL22 had no effect (Fig. 5A). Of the cytokines tested, only IL-1β had a significant effect on CMKLR1 levels at 48h (Fig. 5B) Likewise, CCRL2 and GPR1 were significantly upregulated only by IL1β or IL1β+OSM at the 24h time point, and by IL1β at the 48h time point in the case of CCRL2 (Fig. 5). CCRL2 was also significantly dowregulated by IL22 at 48h, whereas GPR1 expression was not altered (Fig. 5B).

**Fig 6:**
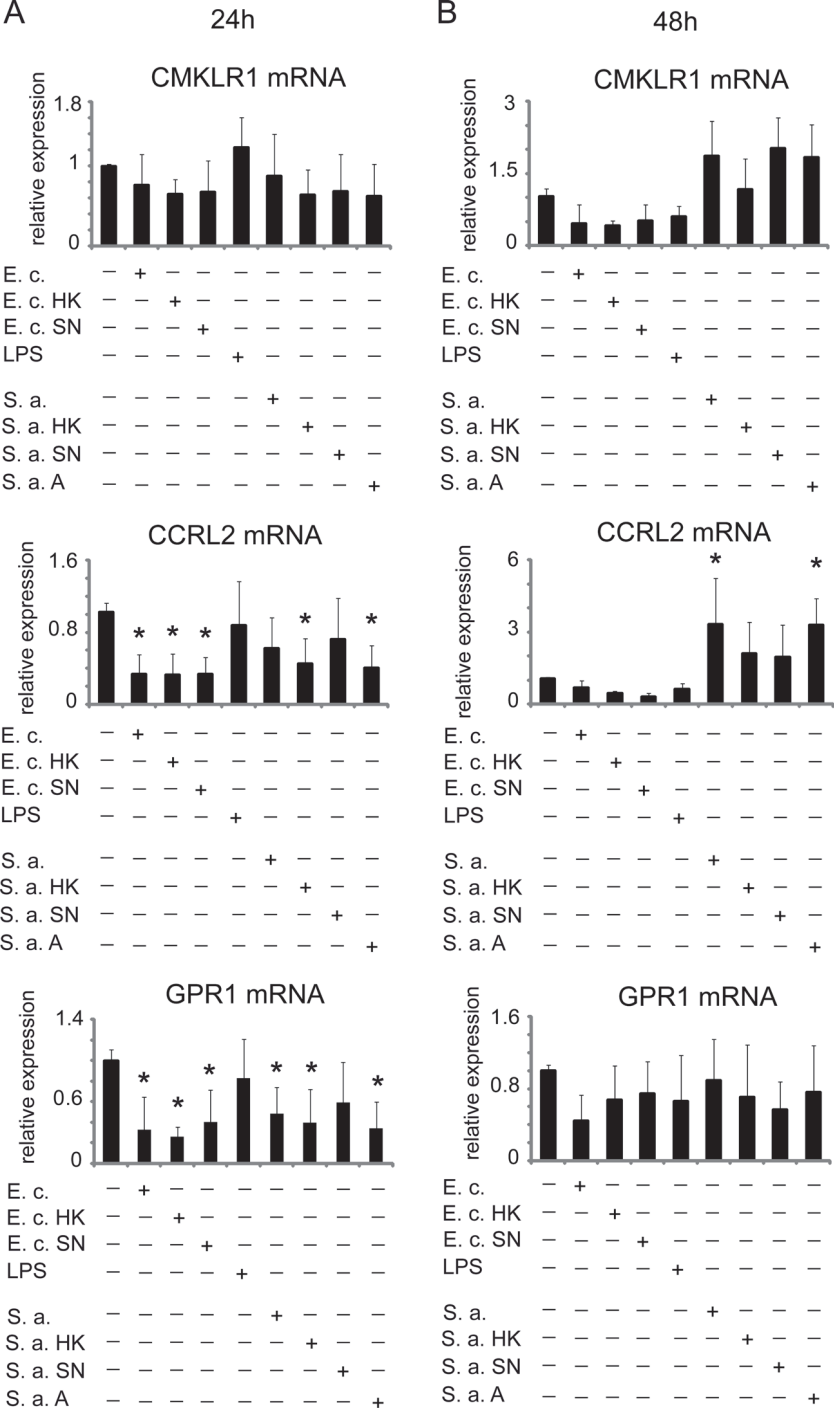
Expression of chemerin receptors in human skin equivalents treated with bacteria. Keratinocytes were treated with the indicated factors for 24h (A) or 48 h (B). RT-QPCR was performed and the expression data were normalized to cyclophilin A and expressed relative to unstimulated cells. Mean ± SD of 5–7 independent experiments is shown. Statistical significance comparing cytokine-treated cells vs. untreated cells (* p<0.05) was determined by ANOVA followed by a Bonferroni post hoc test.

CCRL2 and GPR1 RNA expression was significantly downregulated by 24h-treatment with *E. coli* and *E. coli* products, and to a lesser extent by *S. aureus*, whereas levels of CMKLR1 were unaffected (Fig. 6A). Interestingly, at 48h, CCRL2 expression was significantly induced by live *S. aureus* but not by *E. coli* and its derivatives (Fig. 6B). A similar trend was noted for CMKLR1. Taken together, these data suggest that different regulatory mechanisms underlie the expression of each of the chemerin receptors in human epidermis.

### Regulation of chemerin and its receptors by bacteria in mouse skin in vivo

**Fig 7:**
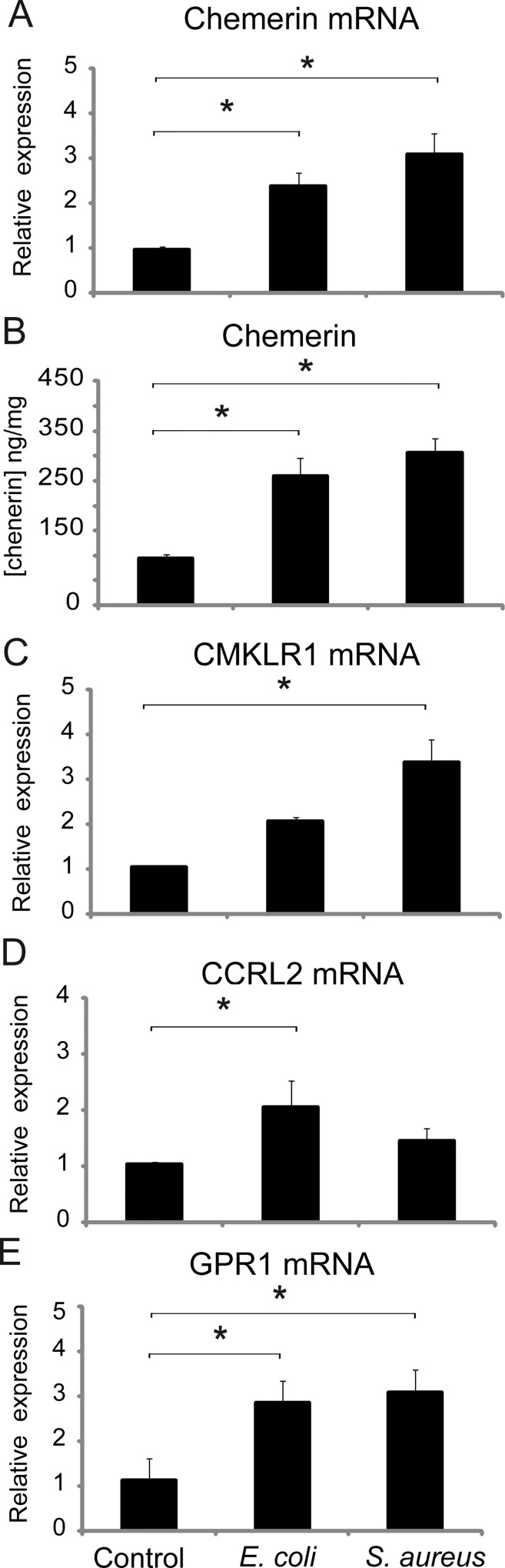
Bacteria controls the expression of chemerin and its receptors *in vivo*. Mice were ectopically treated with *S. aureus, E coli* or PBS (control) for 24h. The skin exposed to the treatment was then collected for RNA and protein isolation. Chemerin and chemerin receptor message was determined by RT-QPCR. The expression data was normalized to cyclophilin A and presented relative to PBS-treated skin (A, C-E). The amount of chemerin in skin lysates, normalized to total protein was determined by ELISA (B). Data are shown as the mean ±SEM from six mice in each group. Statistically significant differences between PBS-treated and bacteria-treated mice is indicated by an asterisk (*, p<0.05; ANOVA followed by a Bonferroni post hoc test).

Due to the pronounced elevation of chemerin levels by bacteria and the differential effects of *E. coli* and *S. aureus* on chemerin receptor expression in human skin equivalents, we next asked if these responses occur *in vivo*. Mice were ectopically treated with *E. coli* or *S. aureus*, and the skin analyzed for chemerin and chemerin receptor expression 24h later. Both *E. coli* or *S. aureus* significantly upregulated chemerin mRNA and chemerin protein levels in skin lysates (Fig. 7A and B). However, similar to human skin equivalents, *S. aureus* seemed to be more potent in inducing chemerin expression compared with *E. coli*, although this trend did not reach statistical significance. *S. aureus* significantly increased CMKLR1 and GPR1 RNA expression in the skin (Fig. 7C and E), while *E. coli* significantly increased the expression of CCRL2 and GPR1 (Fig. 7D &E). Together, these data suggest that the expression of chemerin and its receptors are influenced in distinct fashion by cutaneous microbes.

### Chemerin is required for maximal bactericidal effects in vivo

**Fig 8:**
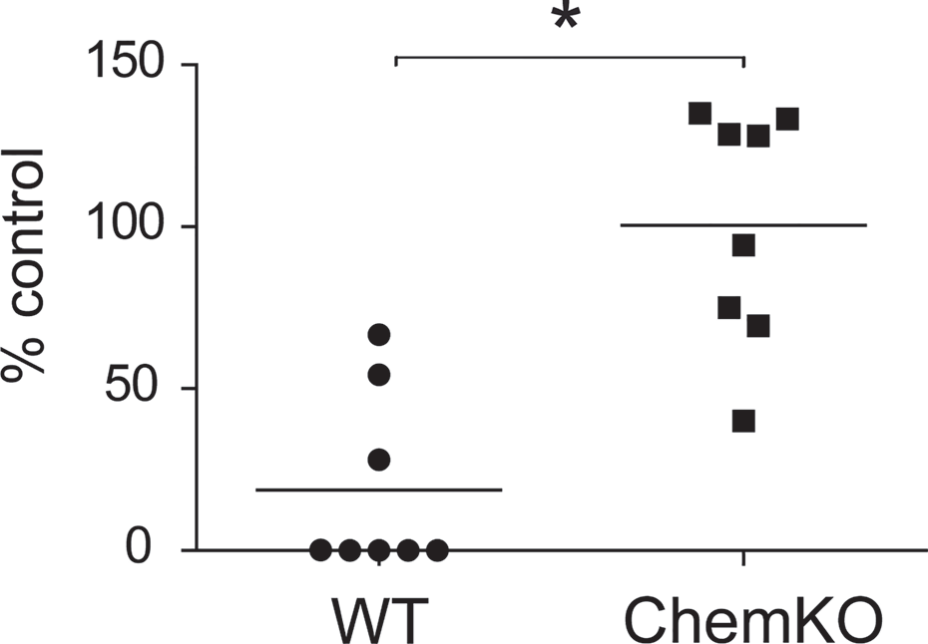
Chemerin is bactericidal *in vivo*. Chemerin–deficient (ChemKO) and WT mice were ectopically treated with *S. aureus*. Bacteria were retrieved from skin 24h later, and presented as a % of input inoculum. Each data point represents one experiment and a horizontal line indicate the mean value in each group. *p<0.05, by *t* test.

Given the significant local induction of chemerin in the skin in response to bacterial challenge, we next asked if chemerin controls bacterial burden in skin. Chemerin-deficient mice and wild type controls were topically infected with *S. aureus*, and the bacterial load recovered from the skin surface 24h later was measured by colony-forming assay. Chemerin-deficient mice harbored at least 10-fold higher bacterial levels compared to WT (Fig. 8). These data suggest that chemerin plays a key role in restricting bacteria growth in skin.

## Discussion

Here we report on previously unappreciated regulators of chemerin synthesis in the epidermis that link chemerin expression to both clinical findings in psoriasis and antimicrobial functions of chemerin in skin.

First, treatment of model epidermis with IL-17 and IL-22 recapitulate the reduction in chemerin levels reported in affected skin from psoriasis patients. Although the nature and significance of chemerin downregulation in lesional psoriatic skin remains obscure, we reasoned that chemerin expression might be affected by the same mediators that drive the disease processes. Genetic studies, usage of therapeutic antagonists, as well as recently developed imiquimod-based mouse model of psoriasis, established a pivotal role for the IL-17 as a driver in skin inflammation in psoriasis [39,45]. In addition, IL-22 has emerged as a key regulator of keratinocyte hyperplasia in this disorder [40,46,47]. Deficiencies in either, IL-17 or IL-22 result in partial protection, whereas absence of both IL-17- and IL-22-mediated responses confers almost total protection against the disease, suggesting additive or synergistic effects of these cytokines in the development of skin changes. Keratinocytes appear to be one of the main targets of IL-17 and IL-22 in psoriatic skin [39,40]. This is supported by the finding that the absence of IL-17 or IL-22 correlates with marked reduction in epidermal thickening along with diminished numbers of skin infiltrating immune cells *in vivo*. Moreover, keratinocytes respond to these cytokines *in vitro* with a psoriatic-like gene expression signature that includes production of proinflammatory cytokines, chemokines, complement components and antimicrobial peptides [39,40,47]. Our work indicates that chemerin may be a regulatory target of IL-17 and IL-22 in epidermis, potentially influencing skin cell responses in psoriasis.

Second, we identified two different chemerin regulation patterns in response to cytokines that are elevated or induced in psoriatic skin. In contrast to IL-17 and IL-22, which suppressed chemerin expression, OSM and IL-1β significantly increased chemerin production, despite the fact that all four cytokines are potent keratinocyte activators with potential roles in the pathology of psoriasis [38,43,48]. IL-1β has been assigned a prominent function in various aspects of cutaneous inflammation, for example, as a key contributing factor to the development and maturation of IL-17 secreting T cells, or in the recruitment of neutrophils to psoriatic skin [49,50,51]. On the other hand, OSM was linked to the pathology of psoriasis through its ability to inhibit expression of keratinocyte differentiation markers, including filaggrin and loricrin, which are decreased in the skin of psoriatic patients, or through inducing AMPs in reconstituted epidermis, such as psoriasin (S100A7), calgranulin C (S100A12) and β-defensin 2, which are strongly associated with psoriasis [38,43,52]. Although these OSM-mediated skin alterations suggest a pathogenic role of OSM in the disease, this cytokine may also contribute to attenuating the pathology, depending, for example, on the phase of the disease. This is supported by its well-defined role as an acute phase mediator as well as the observation that in reconstituted epidermis, OSM also downregulated sets of genes regarded as pro-inflammatory in psoriasis, such as Th1-type signaling molecules [43]. The opposing effects of OSM and IL-1β compared with IL-17 and IL-22 on chemerin production in keratinocytes suggests different roles for the former in regulating chemerin-mediated skin changes. Notably, in contrast to IL-17 and IL-22, which had no effect or downregulated the chemerin receptors, IL-1β and to the lesser extend OSM increased expression of the receptors, suggesting that chemerin might have a particularly strong impact on skin pathophysiology when IL-1β and/or OSM are present. Since the epidermal disruption that occurs in psoriasis may lead to a compensatory engagement of cytokines involved in restoration of homeostasis, such as acute phase mediators-OSM and IL-1, chemerin and chemerin receptor levels that rise in response to OSM and IL-1β may serve to improve skin conditions.

Third, our findings indicate that the epidermis is a functional bacteria-responsive anatomic site for chemerin production. The major function of the epidermis is to provide a barrier against the external environment that includes a variety of pathogenic microorganisms. Our data suggest that keratinocytes respond to microbial stimuli with chemerin synthesis. They also indicate that the epidermis, through upregulation of CCRL2 or CMKLR1, is likely to respond to chemerin in an autocrine manner when challenged by specific bacteria strains. Whereas *E. coli* and *S. aureus* both increased chemerin expression in human skin equivalents *in vitro* as well as mouse skin *in vivo*, chemerin receptor expression appeared to be differentially regulated by these bacteria strains. Most striking was a stimulatory role of *S. aureus* but not *E. coli* on CCRL2 expression in human skin equivalents. Restricting keratinocyte response to upregulation of chemerin but not CMKLR1 or CCRL2, as was the case for *E. coli*-mediated stimulation, may be a mechanism that diminishes CCRL2-mediated accumulation of chemerin on keratinocyte surfaces or CMKLR1-mediated signaling in keratinocytes, allowing free chemerin to act as an AMP. In contrast, *S. aureus* has the potential to contribute to epidermal biology by virtue of its reciprocal induction of chemerin and chemerin receptor expression. Whereas the secretion of chemerin by *S. aureus*-stimulated keratinocytes may contribute to establishing a biochemical shield to microbial colonization of skin by other bacteria, upregulation of chemerin receptors might foster chemerin-mediated, yet-to- be-identified functional changes in mammalian skin.

*S. aureus* and *E. coli* are likely to deploy various mechanisms to affect production of chemerin and chemerin receptors in keratinocytes. These may include soluble factors and/or non-secreted bacterial components, such as structures of the bacterial wall that differ substantially between these two microorganisms. Killing of either bacteria with heat, diminished chemerin production in keratinocytes, suggesting that bacteria viability is an important determinant associated with chemerin synthesis. A new concept has emerged that the recognition of so-called vita-PAMPs (viability associated pathogen-associated molecular patterns) that are present only in viable bacteria elicits unique responses [53]. These include bacterial messenger RNA. The stimulation of chemerin production by vita-PAMPs may explain the differential potency of live and dead bacteria to regulate chemerin expression in keratinocytes. Since chemerin synthesis in reconstituted human epidermis is also triggered to some extent by bacterial supernatants, soluble factors may also be involved in promoting chemerin synthesis in keratinocytes.

Together, our findings reveal an inherent ability of human and mouse epidermis to express high levels of chemerin. Our previous work demonstrated the potent antimicrobial activity of human keratinocyte-derived chemerin [25], and our present study shows substantially diminished antimicrobial activity in chemerin-deficient mice. Thus, elevation of chemerin levels by acute phase cytokines and specific bacteria strains, and downregulation by cytokines associated with psoriasis may reflect a programmed response to skin challenge that regulates defensive functions of this organ.
